# Comparison of PSMA-TO-1 and PSMA-617 labeled with gallium-68, lutetium-177 and actinium-225

**DOI:** 10.1186/s13550-022-00935-6

**Published:** 2022-10-01

**Authors:** Catherine Meyer, Vikas Prasad, Andreea Stuparu, Peter Kletting, Gerhard Glatting, Jonathan Miksch, Christoph Solbach, Katharina Lueckerath, Lea Nyiranshuti, Shaojun Zhu, Johannes Czernin, Ambros J. Beer, Roger Slavik, Jeremie Calais, Magnus Dahlbom

**Affiliations:** 1 Ahmanson Translational Theranostics Division, Department of Molecular and Medical Pharmacology, David Geffen School of Medicine, UCLA, 650 Charles E Young Drive South, Los Angeles, CA 90095-7370 USA; 2 Department of Nuclear Medicine, University Hospital Ulm, Ulm, Germany; 3 Atreca, Inc., South San Francisco, CA USA; 4 Clinic for Nuclear Medicine, University Hospital Essen, Essen, Germany

**Keywords:** PSMA-TO-1, PSMA-617, Prostate cancer, Radioligand therapy, Dosimetry

## Abstract

**Background:**

PSMA-TO-1 (“Tumor-Optimized-1”) is a novel PSMA ligand with longer circulation time than PSMA-617. We compared the biodistribution in subcutaneous tumor-bearing mice of PSMA-TO-1, PSMA-617 and PSMA-11 when labeled with ^68^Ga and ^177^Lu, and the survival after treatment with ^225^Ac-PSMA-TO-1/-617 in a murine model of disseminated prostate cancer. We also report dosimetry data of ^177^Lu-PSMA-TO1/-617 in prostate cancer patients.

**Methods:**

First, PET images of ^68^Ga-PSMA-TO-1/-617/-11 were acquired on consecutive days in three mice bearing subcutaneous C4-2 xenografts. Second, 50 subcutaneous tumor-bearing mice received either 30 MBq of ^177^Lu-PSMA-617 or ^177^Lu-PSMA-TO-1 and were sacrificed at 1, 4, 24, 48 and 168 h for ex vivo gamma counting and biodistribution. Third, mice bearing disseminated lesions via intracardiac inoculation were treated with either 40 kBq of ^225^Ac-PSMA-617, ^225^Ac-PSMA-TO-1, or remained untreated and followed for survival. Additionally, 3 metastatic castration-resistant prostate cancer patients received 500 MBq of ^177^Lu-PSMA-TO-1 under compassionate use for dosimetry purposes. Planar images with an additional SPECT/CT acquisition were acquired for dosimetry calculations.

**Results:**

Tumor uptake measured by PET imaging of ^68^Ga-labeled agents in mice was highest using PSMA-617, followed by PSMA-TO-1 and PSMA-11. ^177^Lu-PSMA tumor uptake measured by ex vivo gamma counting at subsequent time points tended to be greater for PSMA-TO-1 up to 1 week following treatment (*p* > 0.13 at all time points). This was, however, accompanied by increased kidney uptake and a 26-fold higher kidney dose of PSMA-TO-1 compared with PSMA-617 in mice. Mice treated with a single-cycle ^225^Ac-PSMA-TO-1 survived longer than those treated with ^225^Ac-PSMA-617 and untreated mice, respectively (17.8, 14.5 and 7.7 weeks, respectively; *p* < 0.0001). Kidney, salivary gland, bone marrow and mean ± SD tumor dose coefficients (Gy/GBq) for ^177^Lu-PSMA-TO-1 in patients #01/#02/#03 were 2.5/2.4/3.0, 1.0/2.5/2.3, 0.14/0.11/0.10 and 0.42 ± 0.03/4.45 ± 0.07/1.8 ± 0.57, respectively.

**Conclusions:**

PSMA-TO-1 tumor uptake tended to be greater than that of PSMA-617 in both preclinical and clinical settings. Mice treated with ^225^Ac-PSMA-TO-1 conferred a significant survival benefit compared to ^225^Ac-PSMA-617 despite the accompanying increased kidney uptake. In humans, PSMA-TO-1 dosimetry estimates suggest increased tumor absorbed doses; however, the kidneys, salivary glands and bone marrow are also exposed to higher radiation doses. Thus, additional preclinical studies are needed before further clinical use.

**Supplementary Information:**

The online version contains supplementary material available at 10.1186/s13550-022-00935-6.

## Introduction

The prostate-specific membrane antigen (PSMA) is highly expressed by prostate cancer (PCa) cells and is a relevant target for PCa imaging and therapy. Radioligand therapy (RLT) using PSMA-targeting ligands is an emerging therapeutic option in men with metastatic castration-resistant prostate cancer (mCRPC). However, more than half of mCRPC patients treated with PSMA RLT eventually fail therapy [1, 2]. Reasons for disease progression or patient relapse may include insufficient radiation dose delivery (due to low PSMA expression, insufficient administered activity and insufficient tumor retention time of the RLT agent) or radio resistance (tumor biology, germline or somatic mutations, DNA damage repair mechanisms) [3–8]. One potential strategy to increase tumor radiation doses is to extend the PSMA ligand circulation time. Reducing the blood clearance (by the kidneys and other off-target organs) can potentially lead to an increased tumor accumulation of the radiolabeled peptide. One approach using an Evans blue albumin-binding moiety was shown to yield a twofold–sixfold increase in the number of disintegrations in tumors using ^177^Lu-EB-PSMA-617 compared to ^177^Lu-PSMA-617 [9]. This Evans blue modification approach has also been investigated for peptide receptor radionuclide therapy which was shown to increase tumor dose relative to ^177^Lu-DOTATATE; and, in a subsequent phase I clinical trial, improved response rates (NCT03478358) [10, 11].

**Fig. 1:**
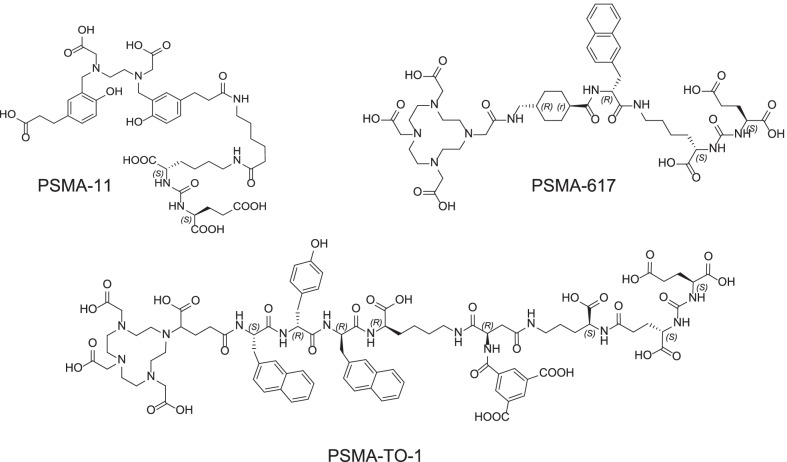
PSMA-targeting peptides. Chemical structures for PSMA-11, PSMA-617 and PSMA-TO-1 used in this work. PSMA-TO-1 contains an extended linker with additional naphthyl groups

In this study, we investigated a novel PSMA ligand called PSMA-TO-1 (“Tumor-Optimized-1”) that was developed for prolonged circulation time and higher tumor uptake (Dr. H.-J. Wester, Technische Universität München, Germany). In comparison with PSMA-617 and PSMA-I&T, PSMA-TO-1 contains an extended linker with additional naphthyl groups to increase albumin and other protein bindings in blood, thereby increasing the lipophilicity (chemical structures shown in Fig. 1). Experimentally, PSMA-TO-1, also known as PSMA-71, was shown to improve internalization and exhibit greater albumin binding than both PSMA-617 and PSMA-I&T (98 vs. 74 and 78%, respectively) [12]. Additionally, in mice bearing LNCaP tumors, biodistribution data of ^177^Lu-PSMA-TO-1 revealed threefold greater tumor uptake 24 h post-injection compared with ^177^Lu-PSMA-I&T (14.3 ± 0.9 vs. 4. 1 ± 1.1%IA/g) with an improved tumor-to-kidney uptake ratio (0.4 vs. 0.1, respectively) [12]. These promising findings warranted further exploration into the performance and application of PSMA-TO-1.

Here we directly compared preclinically PSMA-TO-1 as an imaging and therapeutic agent with the two most widely studied PSMA ligands: PSMA-11 and PSMA-617. First, we determined the biodistribution of the diagnostic compounds ^68^Ga-PSMA-TO-1/-617/-11 in murine models. Second, we assessed ex vivo the biodistribution of the therapeutic compounds ^177^Lu-PSMA-TO-1 and ^177^Lu-PSMA-617 in a subcutaneous xenograft model. Third, we conducted a murine survival study in a model of disseminated prostate cancer to compare the therapeutic efficacy of ^225^Ac-PSMA-TO-1 and ^225^Ac-PSMA-617. Additionally, we report the dosimetry data of ^177^Lu-PSMA-TO-1 in humans.

## Methods

### Study design

This work was investigator-initiated and self-funded. Preclinical experiments were conducted at UCLA (USA). Clinical procedures were performed at Ulm University (Germany). Preclinical and clinical procedures were conducted independently and separately. The clinical and preclinical studies were not planned as a translational study and were merged retrospectively into a single report.

### Preclinical experiments

#### Cell culture

The human-derived, PSMA-expressing, castration-resistant prostate cancer tumor cell line C4-2 was used for all preclinical work (courtesy Dr. G. Thalmann; Department of Urology, Inselspital Bern, Switzerland). Cells were maintained in Roswell Park Memorial Institute 1640 medium supplemented with 10% fetal bovine serum (Omega Scientific) at 37 °C and 5% CO_2_. They were monitored regularly for mycoplasma contamination using the Venor GeM Mycoplasma Detection Kit (Sigma-Aldrich) and authenticated by short tandem repeat sequencing (August 2019; Laragen). The parental cells were engineered to express firefly luciferase (C4-2-luc) by transduction with an amphotropic retrovirus encoding enhanced firefly luciferase followed by fluorescence-activated cell sorting of transduced cells.

#### Animal studies

Immunodeficient, 6–8-weeks-old NOD SCID gamma (NSG) male mice (Charles River Labs, Wilmington, MA) were housed under pathogen-free conditions with food and water ad libitum, and a 12–12 h light–dark cycle. Veterinarian staff and investigators observed the mice daily to ensure animal welfare and determine if humane endpoints (e.g., hunched and ruffled appearance, apathy, ulceration, severe weight loss and tumor burden) were reached. All animal studies were approved by the UCLA Animal Research Committee (# 2005–090).

#### Radiopharmaceutical synthesis

PSMA-11 and PSMA-617 precursors were obtained from ABX advanced biochemical compounds (Radeberg, Germany). PSMA-TO-1 precursor was obtained from Dr. H.-J. Wester (Technische Universität München, Germany). Gallium-68 was eluted from an Eckert & Ziegler IGG100 Generator and the peptide precursors (5 nmol for PSMA-11 and PSMA-TO-1; 10 nmol for PSMA-617) were labeled with ^68^Ga to obtain 19–24 mCi of final products according to previously published protocols [13].

No-carrier-added ^177^LuCl_3_ was obtained from Spectron MRC and PSMA-617 and PSMA-TO-1 were radiolabeled as previously described with a molar activity of 84 GBq/µmol [14]. Actinium-225 was supplied by the Isotope Program within the Office of Nuclear Physics in the Department of Energy’s Office of Science and radiolabeled as previously described (molar activity of 130 MBq/µmol) [13].

#### ^68^Ga-PSMA-TO-1/-617/-11 PET/CT imaging in mice

NSG mice bearing subcutaneous C4-2 tumors underwent PET/CT imaging with each of the following compounds on consecutive days: ^68^Ga-PSMA-11, ^68^Ga-PSMA-617, and ^68^Ga-PSMA-TO-1 in the same 3 mice. Average tumor volumes over the 3 days were 660 ± 35 mm^3^, 190 ± 32 mm^3^ and 243 ± 1.5 mm^3^ for mouse 1, 2 and 3, respectively. PET images were acquired 60 min after intravenous administration of 1.1 MBq ^68^Ga-PSMA in 100 µL volume (PSMA-TO-1 on day 1; PSMA-11 on day 2; PSMA-617 on day 3) using the preclinical Genisys 8 PET/CT scanner (Sofie Biosciences). Attenuation-corrected images were reconstructed using maximum likelihood expectation–maximization with 60 iterations. The following parameters were applied for CT imaging: 40 kVp, 190 mA, 720 projections, and 55-ms exposure time per projection. The resulting PET/CT images were analyzed for tumor volume and percent injected activity uptake per gram using VivoQuant Imaging Software (Invicro, Boston, MA). One-way ANOVA with Bonferroni’s multiple comparisons test was used to compare the tumor uptake of each ligand.

#### ^177^Lu-PSMA-TO-1/-617 ex vivo biodistribution study in mice

NSG mice were subcutaneously inoculated into the right shoulder region with 5 million C4-2 cells in 100 µl matrigel (*n* = 50 mice). After 3 weeks, when the average tumor size was approximately 300 mm^3^, mice were randomized based on tumor volume and treated with either 30 MBq of ^177^Lu-PSMA-617 or ^177^Lu-PSMA-TO-1 (*n* = 25 mice PSMA-617; *n* = 25 mice PSMA-TO-1). Treatment activity (30 MBq) was selected based on previous studies [14]. Following treatment, mice were sacrificed at 5 time points (*n* = 5 mice/time point): 1, 4, 24, 48 and 168 h (7 days). The following organs were collected and weighed prior to gamma counting for activity quantification with ^177^Lu detection energy window of 189–231 keV (Cobra II Auto-Gamma; Packard Instrument Co.): blood, tumor, submandibular salivary glands, heart, lungs, liver, bilateral kidneys, spleen, stomach (with contents), intestines (with contents), prostate, testes, muscle, femur (with and without bone marrow), bone marrow and the brain. Kidney organ doses were estimated from the measured uptake values using the 25 g mouse model in OLINDA/EXM version 2.2.0 [15]. The kidney activity data were fit and integrated to yield residence times and kidney self-doses. The multiple t test method was used for biodistribution statistical comparisons and the Holm–Sidak method was applied to determine statistical significance (set to ≤ 0.05).

#### ^225^Ac-PSMA-TO-1/-617 survival study in mice

NSG mice underwent intracardiac inoculation with 500,000 C4-2-luc cells (in 50 µl PBS) to achieve widespread microscopic visceral and bone metastases, as previously described (*n* = 25 mice) [13]. After 5 weeks, mice were randomized into 3 groups: treatment with ^225^Ac-PSMA-617 (*n* = 10), treatment with ^225^Ac-PSMA-TO-1 (*n* = 10), or untreated controls (*n* = 5). Before treatment, the whole body tumor burden was assessed by bioluminescence imaging (IVIS Lumina III in vivo imaging system, Perkin Elmer). Treatment groups comprised equal proportions of mice with higher and lower tumor burden to create groups with comparable average tumor burden (Additional file 1: Figure S1). Two days prior to treatment, the mean bioluminescence radiance for non-treated, ^225^Ac-PSMA-617-, and ^225^Ac-PSMA-TO-1-treated mice was 2.23 × 10^9^ ± 1.46 × 10^9^ p/sec/cm2/sr (*n* = 5), 2.07 × 10^9^ ± 2.09 × 10^9^ (*n* = 10), and 2.37 × 10^9^ ± 1.56 × 10^9^ (*n* = 10), respectively (not significantly different; *p* > 0.42 for all group comparisons). The treatment activity was selected as 40 kBq ^225^Ac for both the PSMA-TO-1 and 617 groups based on previous studies [16]. Mice were sacrificed when they exhibited severe weight loss and showed signs of deteriorating health such as hunching, dehydration, and labored breathing. The overall condition of the animals was assessed using the body conditioning score [17]. A drop in score from 3 (well-conditioned mouse) to 2 (under-conditioned; segmentation of vertebral column evident, dorsal pelvic bones palpable) warranted euthanasia. The Log-rank (Mantel–Cox) statistical test was used for survival analysis (GraphPad Prism 8).

### Clinical procedures

#### Patients

This was a retrospective evaluation of three patients with mCRPC. All patients had end-stage progressive mCRPC disease after all conventional therapies had failed and were referred by their treating uro-oncologist for ^177^Lu-PSMA therapy under compassionate use in compliance with the German Medicinal Products Act, AMG §13 (2b). All three patients gave written informed consent to undergo ^68^Ga-PSMA PET/CT scans and potential ^177^Lu-PSMA-TO-1 therapy after multiple time point dosimetry imaging following national regulations, the updated Declaration of Helsinki, and Good Clinical Practice (GCP). The radiopharmaceuticals were produced in accordance with the German Medicinal Products Act §13(2b) and the responsible regulatory bodies.

#### Radiosynthesis of ^68^Ga-PSMA-11 and ^68^Ga-PSMA-TO-1 for clinical application

PSMA-11 precursor was obtained from ABX advanced biochemical compounds (Radeberg, Germany). PSMA-TO-1 precursor was obtained from Dr. H.-J. Wester (Technische Universität München, Germany). For the production of ^68^Ga-radiopharmaceuticals for clinical use, a 50 mCi iThemba ^68^Ge/^68^Ga-generator (iThemba LABS, South Africa) was used. ^68^Ga-PSMA-11 and ^68^Ga-PSMA-TO-1 were synthesized in a fully automated (Scintomics Gallelut radiosynthesizer) and GMP-compliant process adapted from the data presented previously by Eder et al. [18] by use of 20 µg PSMA-11 precursor (^68^Ga-PSMA-11) and 80 µg PSMA-TO-1 precursor (^68^Ga-PSMA-TO-1), respectively.

#### ^177^Lu-PSMA-TO-1 human dosimetry

Lutetium-177 was acquired from ITM (Garching, Germany) and radiolabeling was performed according to the established procedure in a GMP-certified radiopharmacy laboratory. All three patients were injected with 500 MBq of ^177^Lu-PSMA-TO-1; a sub-therapeutic activity for dosimetry calculation purposes. Planar images were acquired at 1–2 h, 3–4 h, 18–24 h, 42–48 h and 90–164 h with an additional SPECT/CT image acquisition at 18–24 h. The first image was used to determine the 2D calibration factor; planar images from 3 to 48 h aided in identifying the peak in tumor tissue uptake; finally, planar images from 90 to 164 h were used to measure late-phase kinetics. Blood samples were drawn at regular intervals: 10 min, 1 h, 4 h, 24 h and 48 h. Time–activity data were derived from the images and blood samples using NUKDOS software [19]. The image acquisition was performed on a Siemens Symbia T2 SPECT/CT scanner equipped with medium energy parallel-hole collimators. Measurements were done with an energy window between 190 and 225 keV. SPECT was acquired with 30 projections per head on a body contour trajectory with a 128 × 128 matrix and a 30 s acquisition duration per projection. Attenuation correction was based on a low-dose CT scan. SPECT was reconstructed iteratively using the ordered subsets expectation maximization algorithm with 8 iterations, 15 subsets, and a post-reconstruction Gaussian filtering with a filter size of 12 mm. All corrections were executed according to MIRD Pamphlets No. 16 and 26 [20, 21]. For each patient, the kidney with the highest uptake and two tumor lesions were segmented manually. The quantification for the SPECT/CT system was performed using phantom-derived calibration factors with a NEMA phantom that yielded a camera sensitivity of 678 cpm/MBq. The pharmacokinetic information was obtained from the 2D images. For each data set, an exponential fit function was selected based on visual inspection, the standard error, the correlation matrix, and the Akaike information criterion (AIC) [19]. The settings for the measurement error were model-based and a fractional measurement error of 10% was assumed for blood, total body, and kidney, and 15% for tumors [19].

Dosimetry of ^177^Lu-PSMA-617 was also performed 7 days after the ^177^Lu-PSMA-TO-1 administration in one patient (patient #03), using 500 MBq of ^177^Lu-PSMA-617 and a similar method as outlined above. Images were acquired at 1 h, 4 h, 24 h and 48 h, with blood samples taken at 10 min, 1 h, 4 h, 24 h and 48 h, taking into account the remaining activity of ^177^Lu-PSMA-TO-1. The latest imaging time point was set to 48 h since the late-phase kinetics for PSMA-617 are known, and to reduce patient burden. No subsequent therapy with ^177^Lu-PSMA-TO-1 was performed.

#### ^68^Ga-PSMA-TO-1 human PET/CT human images

^68^Ga-PSMA-TO-1 PET/CT was performed in one patient (patient #01). ^68^Ga- PSMA-TO-1 PET/CT images were acquired at 60 min and 120 min after injection of 180 MBq of ^68^Ga-PSMA-TO-1.

## Results

### Preclinical studies

#### ^68^Ga-PSMA-TO-1/-617/-11 preclinical PET/CT imaging

**Fig. 2:**
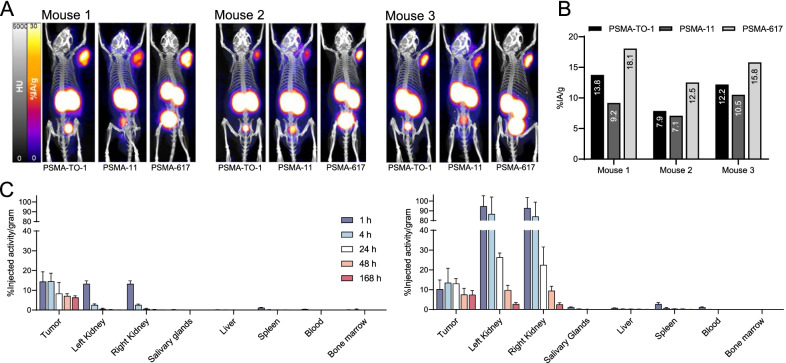
^68^Ga-PSMA PET and ^177^Lu-PSMA biodistribution in mice. **A** Mice bearing subcutaneous C4-2 tumors were imaged 1 h after administration of ^68^Ga-PSMA-TO-1/-11/-617 on consecutive days in the same 3 mice. **B** Corresponding tumor percent injected activity per gram (%IA/g). Mean tumor uptake for PSMA-TO-1, PSMA-11 and PSMA-617 was 11.27, 8.92 and 15.46%IA/g, respectively (not statistically different; *p* > 0.06). **C** Non-decay-corrected tumor and organ %IA/g in mice treated with 30 MBq ^177^Lu-PSMA-617 (left) or 30 MBq ^177^Lu-PSMA-TO-1 (right) (*n* = 5 mice/time point per ligand; bar graphs show mean ± SD). All other organs not shown measured %IA/g < 0.4% at their peak and uptake values for all organs are available in Additional file 1: Tables S1 and S2

All ^68^Ga-labeled PSMA-TO-1, PSMA-11, and PSMA-617 PET images showed high tumor accumulation 1 h after tail vein injection (Fig. 2A). Tracer clearance was predominantly via urinary excretion. We observed the greatest tumor uptake (%IA/g) for PSMA-617, followed by PSMA-TO-1 and PSMA-11 in all three mice (Fig. 2B). Mean tumor uptake for PSMA-TO-1, PSMA-11 and PSMA-617 was 11.27, 8.92 and 15.46%IA/g, respectively. The difference in mean tumor uptake between ligands was not statistically significant (*p* > 0.06 for all group comparisons).

#### ^177^Lu-PSMA-TO-1/-617 ex vivo biodistribution study

Ex vivo counts of ^177^Lu-PSMA-617 and ^177^Lu-PSMA-TO-1 revealed predominant uptake in the subcutaneous tumors and kidneys (Fig. 2C). Consistent with the ^68^Ga-PSMA-TO-1/-617 PET imaging findings, tumor uptake at 1 h post-administration tended to be higher for ^177^Lu-PSMA-617 than ^177^Lu-PSMA-TO-1, though it did not reach statistical significance (14.4 vs. 10.2%IA/g; *p* = 0.207). At all subsequent measurement time points, the absolute tumor uptake tended to be higher for PSMA-TO-1 than PSMA-617 (*p* > 0.13 for all time points). However, kidney uptake was also higher (24%IA/g 24 h after administration, compared with 0.54%IA/g using PSMA-617; *p* = 0.0001, *n* = 5 mice [10 kidneys] per time point per compound). Kidney residence times for ^177^Lu-PSMA-617 and ^177^Lu-PSMA-TO-1 were 2.00E-01 and 5.34E00 MBq-h/MBq, respectively. This translates to a 26 times greater effective dose in the kidneys for PSMA-TO-1 compared with PSMA-617 (5.41E01 vs. 1.44E03 mSv/MBq; or, 43 Sv vs. 1.6 Sv for an injected activity of 30 MBq).

#### ^225^Ac-PSMA-TO-1/-617 survival study

**Fig. 3:**
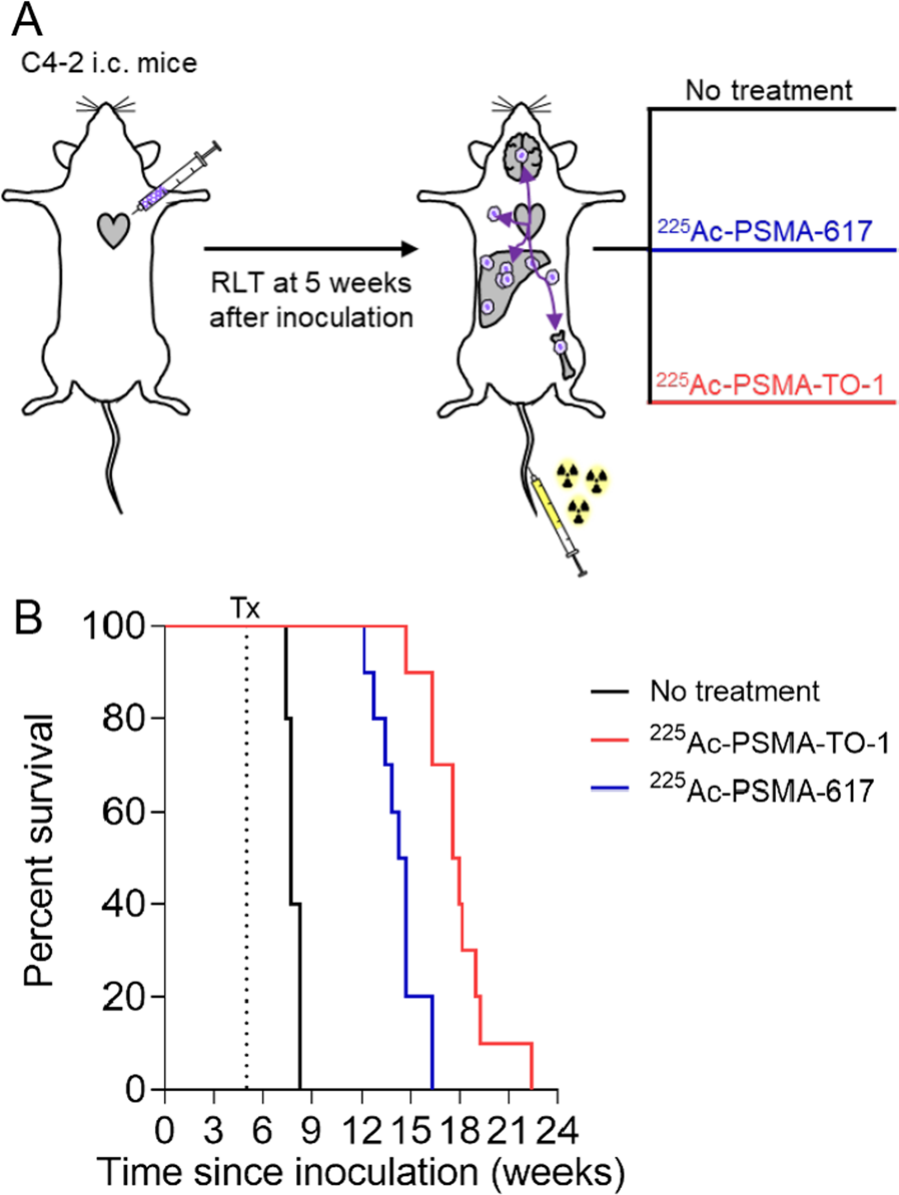
^225^Ac-PSMA-617 and ^225^Ac-PSMA-TO-1 mouse overall survival. **A** Experimental design. **B** Median survival increased from 7.7 weeks (no treatment) to 14.5 weeks for ^225^Ac-PSMA-617 and 17.8 weeks for ^225^Ac-PSMA-TO-1-treated mice (*n* = 10 mice/treatment group and 5 mice for controls: *p* < 0.0001 for NT vs. PSMA-617 or PSMA-TO-1, and *p* = 0.0002 for PSMA-617 vs. PSMA-TO1). RLT = radioligand therapy, i.c. = intracardiac

^225^Ac-PSMA-617 and ^225^Ac-PSMA-TO-1 both significantly prolonged median overall survival relative to untreated mice (7.7 vs. 14.5 and 7.7 vs. 17.8 weeks; *p* < 0.0001). The survival benefit conferred by mice treated with ^225^Ac-PSMA-TO-1 was statistically significant compared to treatment with ^225^Ac-PSMA-617 (*p* = 0.0002) (Fig. 3).

### Patients

The clinical characteristics of the three patients are summarized in Table 1. Of note, patient #01 had multiple liver metastases with low PSMA-expression. Patient #02 had diffuse bone involvement and could not complete the ^177^Lu-PSMA-617 image acquisitions (and therefore dosimetry) because he developed acute renal failure 5 days after administration of 500 MBq of ^177^Lu-PSMA-TO-1. This was likely due to tumor lysis syndrome (see laboratory test in Additional file 1: Figure S2). Despite receiving a sub-therapeutic administered activity, his PSA value decreased after normalization of kidney function (Additional file 1: Figure S2B) and his ECOG performance improved from 3 to 1, suggesting anti-tumor treatment effect.

**Table 1:** Patients’ clinical characteristics

Patient #	Age (years)	PSA (ng/ml)	Prior therapy	miTNM stage
#01	58	320	Bicalutamide enzalutamide, docetaxel, cabazitaxel	T3 N1 M1c (liver)
#02	49	2914	LHRH, bicalutamide enzalutamide, docetaxel, 2 × ^177^Lu-PSMA 617	Tx Nx M1a M1b (bone, lymph nodes)
#03	60	100	Docetaxel, samarium, enzalutamide, 4 × ^177^Lu-PSMA 617	T4 N1 M1a M1b (bone, lymph nodes)

*PSA* prostate-specific antigen, *LHRH* luteinizing hormone-releasing hormone

### ^177^Lu-PSMA-TO-1 human dosimetry

**Fig. 4:**
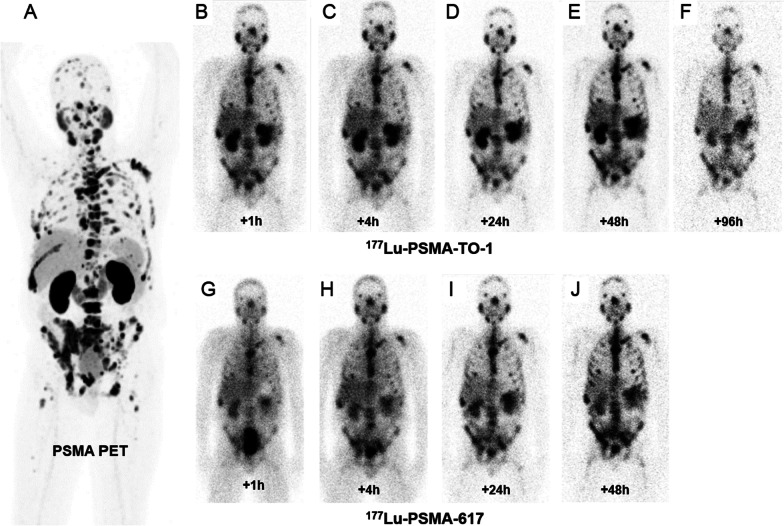
Patient #03. **A** PSMA PET 3D MIP and ^177^Lu-PSMA-TO-1 gamma planar imaging anterior views at + 1 h (**B**), + 4 h (**C**), + 24 h (**D**), + 48 h (**E**), + 96 h (**F**). ^177^Lu-PSMA-617 gamma planar imaging anterior views at + 1 h (**G**), + 4 h (**H**), + 24 h (**I**), + 48 h (**J**). Gamma images are normalized to liver uptake. ^177^Lu-PSMA-TO-1 planar images for patients #01 and #02 are available in Additional file 1: Figures S3 and S4

Kidney, salivary gland, bone marrow and mean ± SD tumor dose coefficients (Gy/GBq) of ^177^Lu-PSMA-TO-1 in patients #01, #02, #03 were 2.5/2.4/3.0, 1.0/2.5/2.3, 0.14/0.11/0.10 and 0.42 ± 0.03/4.45 ± 0.07/1.80 ± 0.57, respectively (Table 2). In patient #03, for whom a dosimetry comparison between PSMA-TO-1 and PSMA-617 could be completed, the therapeutic index (mean tumor dose/critical organ dose) of ^177^Lu-PSMA-617/^177^Lu-PSMA-TO-1 for the kidney, bone marrow and salivary gland was 1.6/0.6, 28.8/18.0 and 0.9/0.8, respectively (Fig. 4). Due to the higher uptake in critical organs, no subsequent radionuclide therapy with ^177^Lu-PSMA-TO-1 was done.

**Table 2:** Clinical dosimetry results

Organ	^177^Lu-PSMA-TO-1 dose coefficients (Gy/GBq)			^177^Lu-PSMA-617 dose coefficients (Gy/GBq)
	Patient #01	Patient #02	Patient #03	Patient #03
Kidneys	2.5	2.4	3.0	0.6
Salivary gland	1.0	2.5	2.3	1.1
Bone marrow	0.14	0.11	0.10	0.033
Tumor 1 Tumor 2	0.40 0.44	4.4 4.5	2.2 1.4	1.10 0.80

### ^68^Ga-PSMA-TO-1 PET/CT human images

**Fig. 5:**
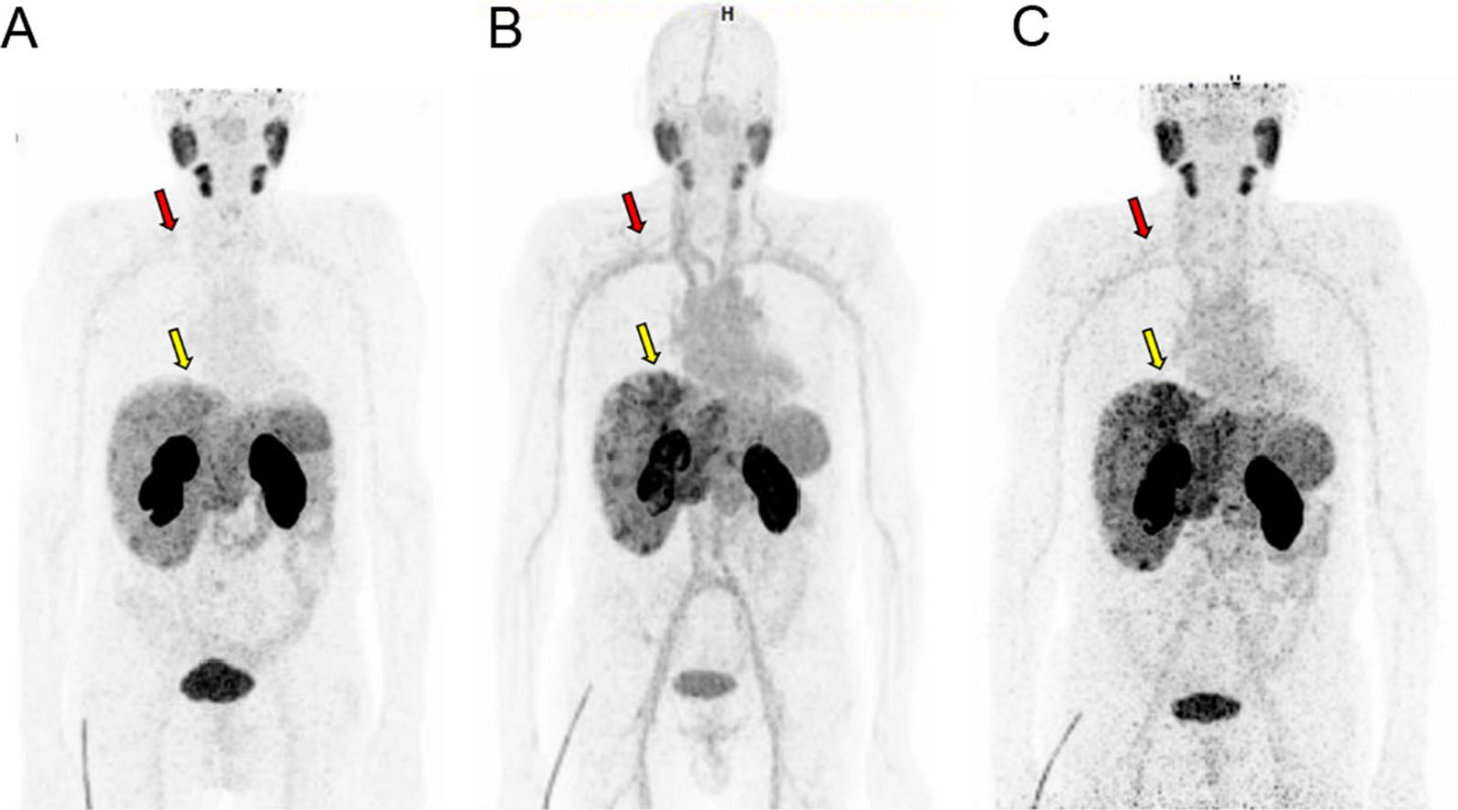
Patient #01 PSMA PET images. **A** ^68^Ga-PSMA-11 PET MIP at + 60 min. **B** ^68^Ga-PSMA-TO-1 PET MIP at + 60 min. **C** ^68^Ga-PSMA-TO-1 PET MIP at + 120 min. Blood pool activity was higher with ^68^Ga-PSMA-TO-1 in comparison with ^68^Ga-PSMA-11 (red arrows). Tumor uptake in liver metastases was slightly higher with ^68^Ga-PSMA-TO-1 in comparison with ^68^Ga-PSMA-11 and increased at + 120 min (yellow arrows, SUVmean 6.0 to 8.0, + 33%). SUVmean values are available in Table 3

For patient #01, who underwent both ^68^Ga-PSMA-TO-1 and ^68^Ga-PSMA-11 PET/CT at 60 min, blood pool activity and kidney uptake were higher with PSMA-TO-1 than with PSMA-11: SUVmean 4.0 vs 1.0 and 30 vs. 14, respectively. Tracer uptake in liver metastases was higher with ^68^Ga-PSMA-TO-1 compared to ^68^Ga-PSMA-11: SUVmean 6.0 vs. 4.0. At 120 min, ^68^Ga-PSMA-TO-1 uptake in metastases increased (SUVmean 8.0, + 33%), whereas blood pool uptake remained constant (see Fig. 5 and Table 3).

**Table 3:** ^68^Ga-PSMA-TO-1 and ^68^Ga-PSMA-11 PET/CT SUVs in patient #01

Organ	SUVmean		
	^68^Ga-PSMA-TO-1 60 min	^68^Ga-PSMA-11 60 min	^68^Ga-PSMA-TO-1 120 min
Blood pool	4	1	4
Spleen	6	6	6
Kidneys	30	14	24
Salivary glands	6	9	10
Liver metastasis	6	4	8

*SUV* standardized uptake value

## Discussion

In this work, we first examined preclinically the biodistribution of 3 PSMA-targeting compounds in tumor-bearing mice. The tumor uptake was higher with PSMA-TO-1 than with PSMA-617 at all measured time points after 1 h. Most notably, this increased tumor uptake was accompanied by murine kidney uptake that was 44 times higher with PSMA-TO-1 than with PSMA-617 24 h after administration, which translates to a 26-fold greater kidney dose in mice. Nevertheless, the preclinical survival study indicated that mice treated with ^225^Ac-PSMA-TO-1 conferred a significant survival benefit compared to those treated with ^225^Ac-PSMA-617 (median overall survival 17.8 vs. 14.5 weeks; *p* = 0.0002). However, long-term nephrotoxic effects could not be studied as the time for histologically measurable parenchymal damage can be greater than 6 months in murine models [22, 23] and euthanasia was required within 7–18 weeks of treatment in all mice.

In humans, we observed the kidney dose coefficients to be 6–8 times higher with ^177^Lu-PSMA-TO-1 than with ^177^Lu-PSMA-617 (2.4–3.0 Gy/GBq in this study vs. 0.39 Gy/GBq for PSMA-617, as published elsewhere [4]). It should be noted that these estimates reflect PSMA-TO-1 doses from 3 patients only, thereby limiting the broad translatability of these results. The kidneys are a primary dose-limiting organ in PSMA-targeted RLT, with commonly used maximum tolerated dose thresholds ranging from 18 to 28 Gy (derived from external beam RT studies) [20, 24]. However, the low dose rate radiation delivered by RLT differs from the high dose rate of external beam RT, and biologically effective doses up to 40 Gy with low dose rate RLT may be well-tolerated [20]. This may in part be explained by the relatively short survival of patients who may not live to experience renal toxicity. Future work may include preclinical approaches to quantify acute kidney damage with molecular or pathological biomarkers as predictors for long-term injury. One study by Pellegrini et al. identified γ-H2AX positive nuclei in the renal cortex, a marker for DNA strand breaks, as a possible indicator for long-term radiation-induced kidney damage [23]. However, meaningful translations of preclinical observations related to renal damage are confounded by the relatively increased radio resistance of murine kidneys compared to humans [25, 26]. Further preclinical studies will be required to better assess the potential nephrotoxic risk of ^177^Lu-PSMA-TO-1 before further clinical use.

We observed reversible renal insufficiency in patient #02, which may be explained by tumor lysis syndrome rather than toxicity. Patient #02 had diffuse bone marrow involvement and developed suspected tumor lysis syndrome one week after receiving only 500 MBq of ^177^Lu-PSMA-TO-1, and 2 weeks after 500 MBq of ^177^Lu-PSMA-617. Tumor lysis syndrome was supported by the subsequent drop in serum PSA levels and normalization of kidney function. This suggests the delivery of high radiation to bone metastases even from a sub-therapeutic administered activity of 500 MBq of ^177^Lu-PSMA-TO-1. However, higher tumor doses may present a potential for treating a patient population with bone or bone marrow lesions. Since PSMA-TO-1 is a longer-circulating peptide, higher bone marrow doses were expected. Indeed, dosimetry data of Patient #03 revealed a threefold higher bone marrow dose with PSMA-TO-1 compared to PSMA-617 (Table 2). Comparing our bone marrow dose in three patients with a larger PSMA-617 cohort [27], the bone marrow dose is 8–tenfold higher for PSMA-TO-1 than with PSMA-617. While this higher dose could pose more risk for hematotoxicity, greater bone marrow exposure and thereby dose delivery may be efficacious to treat patients with bone marrow involvement. At the same time, it should also be considered in the setting of severe bone marrow infiltration whether treatment with a beta- or alpha-emitting radionuclide may be more appropriate. It has been postulated that the shorter tissue penetration range of alpha-emitters, such as ^225^Ac, may be favored in settings where the need to spare surrounding tissue is of greater concern, such as to minimize bone marrow toxicity [28–30].

Finally, another organ of interest in PSMA-targeted RLT is the salivary gland. In this study, the dose coefficient to the salivary glands was two times greater for PSMA-TO-1 compared with PSMA-617 (2.3 vs. 1.1 Gy/GBq in the same patient). The current literature reports salivary gland dose coefficients from ^177^Lu-PSMA-617 treatment ranging from 0.44 and 0.58 Gy/GBq [4] (for submandibular and parotid glands, respectively) to 1.4 Gy/GBq [27]. While xerostomia resulting from ^177^Lu-PSMA RLT is not as frequent nor severe as observed in ^225^Ac-PSMA RLT, the possible clinical significance of increased salivary gland uptake in ^177^Lu-PSMA RLT remains to be characterized. Overall, the higher uptake in normal organs in both preclinical and clinical settings necessitates further preclinical testing and optimization before further clinical use.

## Conclusion

Tumor uptake tended to be greater with PSMA-TO-1 than with PSMA-617 in both preclinical and early clinical settings. Preclinical studies demonstrated a significant survival benefit with ^225^Ac-PSMA-TO-1 over ^225^Ac-PSMA-617. However, a dosimetry comparison of ^177^Lu-PSMA-TO-1 and ^177^Lu-PSMA-617 in one patient suggested that PSMA-TO-1 exposes the kidneys, salivary glands and bone marrow to higher radiation absorbed doses. Before further clinical use, preclinical optimization is required.

## Supplementary Information

**Additional file 1:** Supplementary preclinical and clinical information.

## Data Availability

All preclinical data analyzed during this study are included in this published article and in supplementary materials. Additional clinical data are available from the authors upon reasonable request.

## Abbreviations

AIC: Akaike information criterion
ANOVA: Analysis of variance
CT: Computed tomography
DNA: Deoxyribonucleic acid
ECOG: Eastern Cooperative Oncology Group
IA/g: Injected activity per gram
mCRPC: Metastatic castration-resistant prostate cancer
MIRD: Medical internal radiation dose
NSG: NOD SCID gamma
PET: Positron emission tomography
PSA: Prostate-specific antigen
PSMA: Prostate-specific membrane antigen
RLT: Radioligand therapy
SD: Standard deviation
SPECT: Single-photon emission computed tomography
