# Supplementary material for: Comparison of PSMA-TO-1 and PSMA-617 labeled with gallium-68, lutetium-177 and actinium-225
Source: EJNMMI Res. 2022 Oct 1;12:65. doi: 10.1186/s13550-022-00935-6 (PMC9526774; doi:10.1186/s13550-022-00935-6)
Supplement: Supplementary file 1 — Additional file 1: Supplementary preclinical and clinical information. [file 13550_2022_935_MOESM1_ESM.docx]

**SUPPLEMENTARY MATERIAL**

**TABLES**

**Table 1. ^177^Lu-PSMA-TO-1 biodistribution in organs and tumors (n=5 mice per time point)**

| Organ | %Injected activity/gram (not decay-corrected) | | | | | | | | | |
| --- | --- | --- | --- | --- | --- | --- | --- | --- | --- | --- |
|  | 1 h | | 4 h | | 24 h | | 48 h | | 168 h | |
|  | mean | SD | mean | SD | mean | SD | mean | SD | mean | SD |
| Brain | 0.0423 | 0.0123 | 0.0139 | 0.0036 | 0.0088 | 0.0011 | 0.0051 | 0.0006 | 0.0026 | 0.0003 |
| Tumor | 10.2297 | 4.6313 | 13.4737 | 7.3145 | 13.0771 | 2.4792 | 7.5007 | 3.1617 | 7.4228 | 2.0916 |
| Salivary glands (submandibular) | 1.0885 | 0.2623 | 0.2303 | 0.0865 | 0.1304 | 0.0124 | 0.0979 | 0.0067 | 0.0510 | 0.0086 |
| Heart | 0.7773 | 0.3321 | 0.1448 | 0.0440 | 0.0721 | 0.0060 | 0.0592 | 0.0079 | 0.0296 | 0.0061 |
| Lung | 1.7523 | 0.3634 | 0.3131 | 0.0864 | 0.0888 | 0.0075 | 0.0678 | 0.0132 | 0.0182 | 0.0103 |
| Liver | 0.6795 | 0.1454 | 0.2536 | 0.0313 | 0.2341 | 0.0192 | 0.1904 | 0.0238 | 0.0927 | 0.0156 |
| Left kidney | 94.6983 | 10.7472 | 86.6961 | 17.4208 | 26.3071 | 2.2114 | 9.7597 | 2.4345 | 2.6885 | 0.8252 |
| Right kidney | 92.7447 | 10.8717 | 84.1185 | 14.7831 | 22.4666 | 9.0950 | 9.4479 | 2.2864 | 2.5259 | 0.8554 |
| Spleen | 2.6615 | 0.8513 | 0.5591 | 0.4512 | 0.3423 | 0.0413 | 0.2298 | 0.0991 | 0.0585 | 0.0526 |
| Stomach (with contents) | 0.7389 | 0.4609 | 0.1410 | 0.1924 | 0.5627 | 0.5429 | 0.0555 | 0.0326 | 0.0141 | 0.0128 |
| Intestines (with contents) | 0.2658 | 0.1105 | 0.1115 | 0.0315 | 0.2901 | 0.2198 | 0.0318 | 0.0081 | 0.0552 | 0.0924 |
| Prostate | 0.9544 | 0.1121 | 0.2519 | 0.2471 | 0.0415 | 0.0116 | 0.0185 | 0.0031 | 0.0108 | 0.0050 |
| Muscle | 0.3284 | 0.0814 | 0.0563 | 0.0116 | 0.0187 | 0.0032 | 0.0127 | 0.0024 | 0.0070 | 0.0062 |
| Femur with BM | 1.0550 | 0.5101 | 0.2000 | 0.0683 | 0.1199 | 0.0237 | 0.0901 | 0.0175 | 0.0347 | 0.0250 |
| Femur no BM | 0.6136 | 0.2816 | 0.1543 | 0.0296 | 0.0973 | 0.0470 | 0.0450 | 0.0305 | 0.0298 | 0.0062 |
| Bone marrow | 0.0206 | 0.0061 | 0.0032 | 0.0007 | 0.0012 | 0.0004 | 0.0013 | 0.0008 | 0.0005 | 0.0003 |
| Blood | 1.0301 | 0.3045 | 0.0840 | 0.0380 | 0.0116 | 0.0037 | 0.0035 | 0.0027 | 0.0003 | 0.0003 |
| Testes | 0.7205 | 0.0908 | 0.2266 | 0.0354 | 0.1109 | 0.0419 | 0.0498 | 0.0423 | 0.0444 | 0.0087 |

BM: bone marrow

**Table 2. ^177^Lu-PSMA-617 biodistribution in organs and tumors (n=5 mice per time point)**

| Organ | %Injected activity/gram (not decay-corrected) | | | | | | | | | |
| --- | --- | --- | --- | --- | --- | --- | --- | --- | --- | --- |
|  | 1 h | | 4 h | | 24 h | | 48 h | | 168 h | |
|  | mean | SD | mean | SD | mean | SD | mean | SD | mean | SD |
| Brain | 0.0252 | 0.0050 | 0.0159 | 0.0024 | 0.0125 | 0.0035 | 0.0079 | 0.0016 | 0.0022 | 0.0002 |
| Tumor | 14.392 | 4.9601 | 14.536 | 4.1213 | 8.3500 | 5.6040 | 7.1320 | 1.1532 | 6.3440 | 0.8831 |
| Salivary glands (submandibular) | 0.2440 | 0.0391 | 0.0680 | 0.0192 | 0.0280 | 0.0084 | 0.0180 | 0.0045 | 0.0020 | 0.0045 |
| Heart | 0.2602 | 0.0459 | 0.0262 | 0.0088 | 0.0077 | 0.0014 | 0.0050 | 0.0006 | 0.0007 | 0.0001 |
| Lung | 0.5658 | 0.0681 | 0.0870 | 0.0215 | 0.0216 | 0.0015 | 0.0117 | 0.0013 | 0.0019 | 0.0003 |
| Liver | 0.1560 | 0.0219 | 0.0740 | 0.0167 | 0.0400 | 0.0122 | 0.0300 | 0.0071 | 0.0100 | 0.0000 |
| Left kidney | 13.254 | 1.5517 | 2.5660 | 0.5638 | 0.5500 | 0.3859 | 0.2300 | 0.0678 | 0.0480 | 0.0164 |
| Right kidney | 13.200 | 1.5994 | 2.5440 | 0.5218 | 0.5380 | 0.3350 | 0.2320 | 0.0753 | 0.0500 | 0.0158 |
| Spleen | 1.1740 | 0.1532 | 0.1420 | 0.0879 | 0.0540 | 0.0261 | 0.0460 | 0.0313 | 0.0120 | 0.0045 |
| Stomach (with contents) | 0.0907 | 0.0120 | 0.0793 | 0.0594 | 0.0525 | 0.0311 | 0.0112 | 0.0036 | 0.0009 | 0.0003 |
| Intestines (with contents) | 0.1574 | 0.0357 | 0.1844 | 0.0518 | 0.0594 | 0.0130 | 0.0111 | 0.0037 | 0.0012 | 0.0002 |
| Prostate | 1.2733 | 1.7828 | 0.5901 | 0.8396 | 0.0175 | 0.0262 | 0.0066 | 0.0066 | 0.0013 | 0.0013 |
| Muscle | 0.1970 | 0.1586 | 0.0754 | 0.0993 | 0.0112 | 0.0174 | 0.0022 | 0.0007 | 0.0002 | 0.0000 |
| Femur with BM | 0.2724 | 0.1749 | 0.2464 | 0.1934 | 0.0145 | 0.0035 | 0.0105 | 0.0016 | 0.0034 | 0.0008 |
| Femur no BM | 0.1420 | 0.1195 | 0.0405 | 0.0244 | 0.0072 | 0.0008 | 0.0070 | 0.0017 | 0.0033 | 0.0013 |
| Bone marrow | 0.0860 | 0.0981 | 0.1760 | 0.3601 | 0.0080 | 0.0084 | 0.0140 | 0.0219 | 0.0000 | 0.0000 |
| Blood | 0.4120 | 0.0653 | 0.0200 | 0.0071 | 0.0020 | 0.0045 | 0.0000 | 0.0000 | 0.0000 | 0.0000 |
| Testes | 0.3721 | 0.3075 | 0.0571 | 0.0201 | 0.0272 | 0.0089 | 0.0109 | 0.0054 | 0.0031 | 0.0017 |

BM: bone marrow

**FIGURES**

**
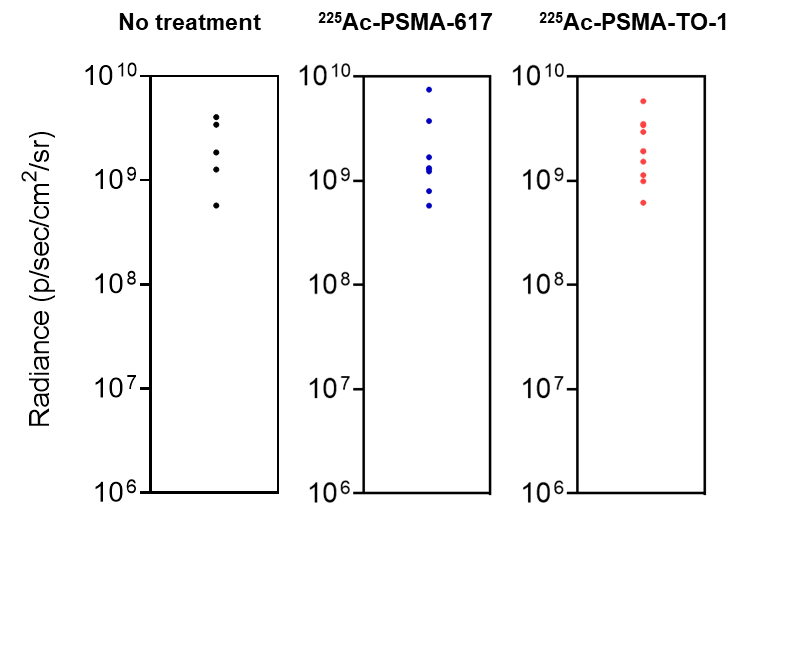
**

**Supplemental Figure 1. Pre-treatment tumor burden in mice.** Whole body tumor burden in mice 2 days prior to treatment with ^225^Ac-PSMA-617/TO-1, as measured by bioluminescence imaging. The mean bioluminescence radiance was 2.23e09 ± 1.46e09 p/sec/cm2/sr (n=5), 2.07e09 ± 2.09e09 (n=10), and 2.37e09 ± 1.56e09 (n=10) for non-treated, ^225^Ac-PSMA-617, and ^225^Ac-PSMA-TO-1 treated mice, respectively (not significantly different; p>0.42 for all group comparisons). The mean radiance across groups was 2.23e09 p/sec/cm2/sr ± 1.48e08.

**
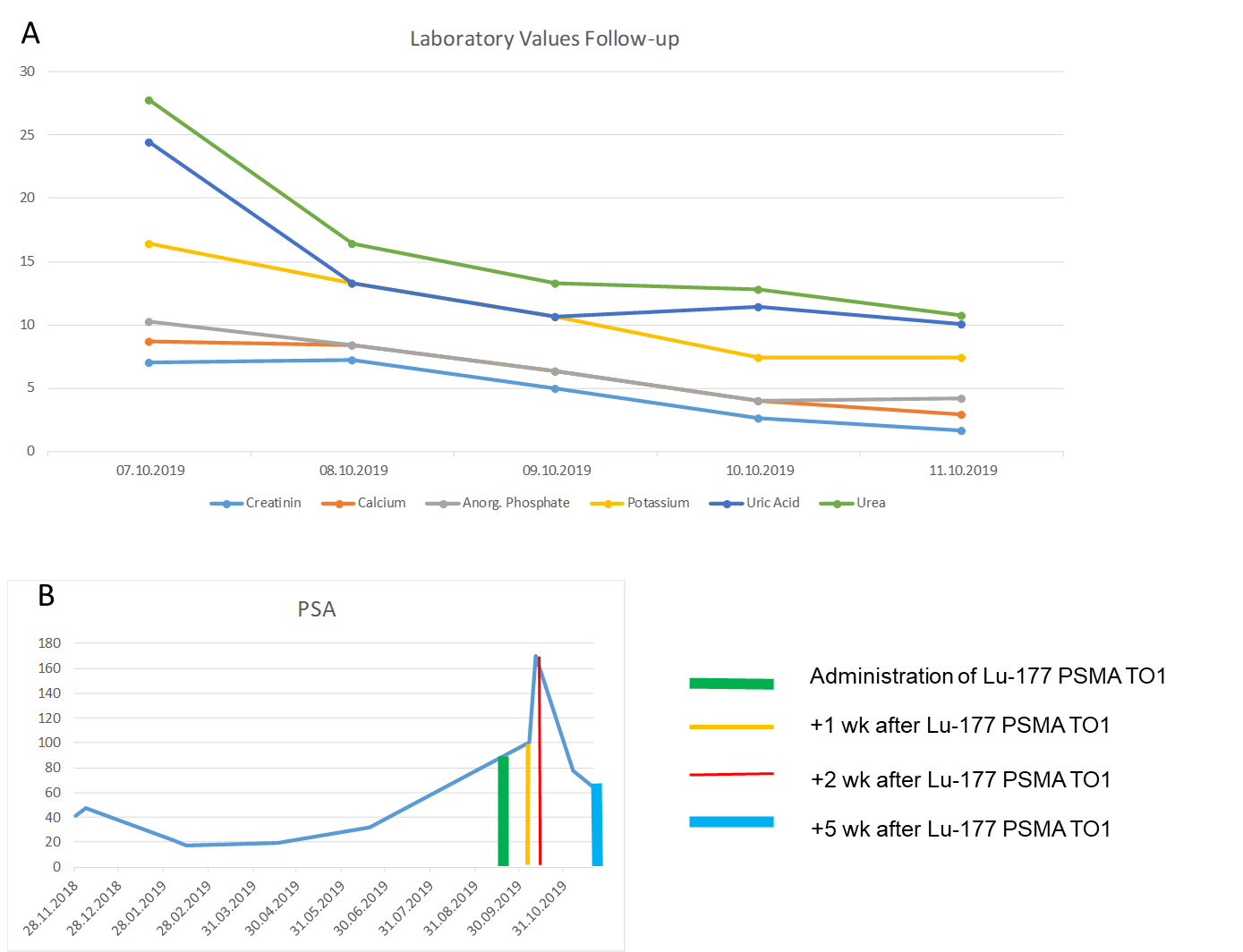
**

**Supplemental Figure 2. Patient #02 laboratory tests results. A)** Laboratory values of patient #02. Normal limits: creatinine 59-104 (for purpose of graphical representation values have been divided by 100), calcium 2.2-2.6, inorganic phosphate 0.81-1.45, potassium 3.4-4.5, uric acid 214-416 (for purpose of graphical representation values have been divided by 100), urea 3-9.2 (for purpose of graphical representation values have been divided by 10). **B)** PSA follow-up. Patient #02 received 500 MBq of ^177^Lu-PSMA-TO-1 on September 30^th^ 2019 and 500 MBq of ^177^Lu-PSMA-617 on October 10^th^ 2019. He developed acute renal insufficiency one week after ^177^Lu-PSMA-TO-1 administration most likely due to tumor lysis syndrome. The patient had diffuse bone marrow carcinomatosis which may be more radiation-sensitive. Despite receiving a sub-therapeutic administered activity, his PSA value decreased after normalization of kidney function and his ECOG performance improved from 3 to 1.

**
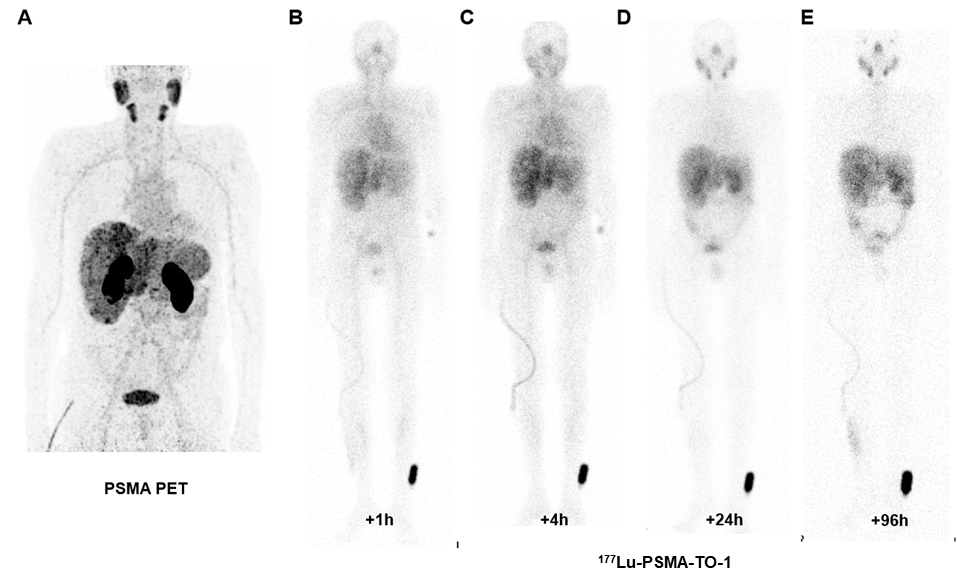
**

**Supplemental Figure 3. Patient #01. A)** PSMA PET 3D MIP and ^177^Lu-PSMA-TO-1 gamma planar imaging anterior views at +1h **(B)**, +4h **(C)**, +24h **(D)**, +96h **(E)**. Gamma images are normalized to liver uptake.

**
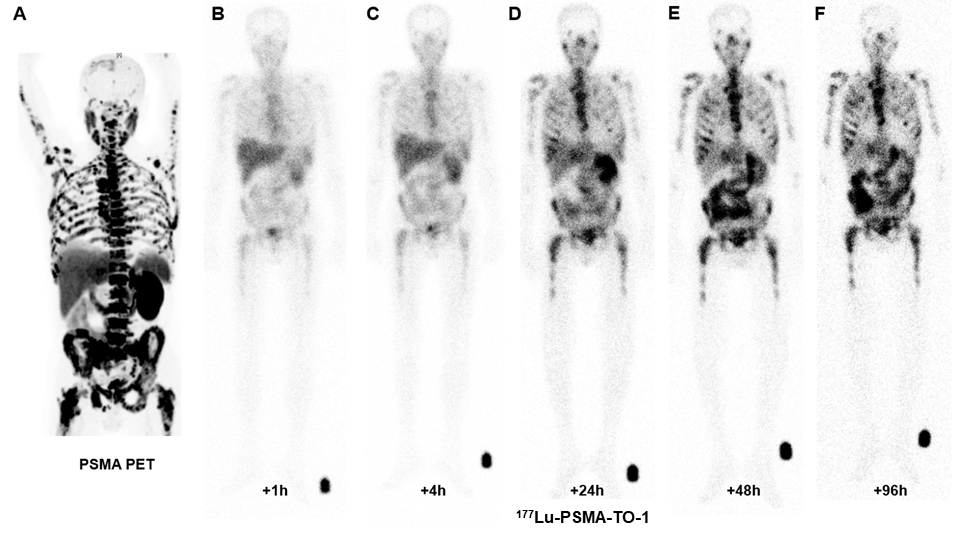
**

**Supplemental Figure 4. Patient #02. A)** PSMA PET 3D MIP and ^177^Lu-PSMA-TO-1 gamma planar imaging anterior views at +1h **(B)**, +4h **(C)**, +24h **(D)**, +48h **(E)** and +96h **(F)**. Gamma images are normalized to liver uptake.
